# A Practical Treatise on Diseases of the Sexual Organs; Adapted to Popular and Professional Reading, and the Exposition of Quackery, Professional and Otherwise

**Published:** 1845-12

**Authors:** 


*A Practical Treatise on Diseases of the Sexual organs: adapted to popular and professional reading, and the exposition of Quackery, professional and otherwise.* By Edward H. Dixon, M. D., etc. etc. 12mo. pp. 260. Burgess & Stringer, New York, 1845.

This is one of the oddest little books we have met with for a long time. It professes to be "adapted to *popular* and *professional* reading;" and is evidently intended for the use of those classes of readers just in the order mentioned—if, indeed, it is expected the latter will read it at all, of which the author expresses the following doubts.—"Neither is it likely this volume will be read by them, ('judicious practitioners') if perchance it should not be entirely condemned from opposition to popular instruction."—"The writer will only add, that though their (the profession's) approbation will be agreeable, their censure will not distress him."

Our opinion of the inutility of popular treatises on practical medicine has been freely expressed on former occasions, and whatever force there may be in the general objection, it becomes yet stronger, in our estimation, when the diseases which are the subjects of discussion in this publication constitute the text of the work. But whatever we may think on this point, there can be no difference of opinion in regard to the fling at the value of professional opinion contained in the last extract. The contempt which it expresses is sufficiently ridiculous, however, to save it from being reciprocated.

The author advocates the doctrine that gonorrhœa and syphilis may originate with individuals without infection; and that in cases, thus arising *de novo*, a specific virus may be secreted, capable of propagating the disease by inoculation or infection. Chancres, he affirms, in the early period, and even after the fifth day, may be cured at once by raising up the indurated parts and clipping them off with a curved scissors. Even in some cases when pain has been felt in the groin, showing the commencement of a bubo, this treatment has succeeded, he says, without the employment of constitutional or any other remedies.

